# Surface Characteristics of Silicon Nanowires/Nanowalls Subjected to Octadecyltrichlorosilane Deposition and n-octadecane Coating

**DOI:** 10.1038/srep38678

**Published:** 2016-12-09

**Authors:** Bekir Sami Yilbas, Billel Salhi, Muhammad Rizwan Yousaf, Fahad Al-Sulaiman, Haider Ali, Nasser Al-Aqeeli

**Affiliations:** 1 Mechanical Engineering Department, King Fahd University of Petroleum & Minerals, Dhahran, Saudi Arabia; 2 Centre of Excellence in Renewable Energy, King Fahd University of Petroleum & Minerals, Dhahran, Saudi Arabia

## Abstract

In this study, nanowires/nanowalls were generated on a silicon wafer through a chemical etching method. Octadecyltrichlorosilane (OTS) was deposited onto the nanowire/nanowall surfaces to alter their hydrophobicity. The hydrophobic characteristics of the surfaces were further modified via a 1.5-μm-thick layer of n-octadecane coating on the OTS-deposited surface. The hydrophobic characteristics of the resulting surfaces were assessed using the sessile water droplet method. Scratch and ultraviolet (UV)-visible reflectivity tests were conducted to measure the friction coefficient and reflectivity of the surfaces. The nanowires formed were normal to the surface and uniformly extended 10.5 μm to the wafer surface. The OTS coating enhanced the hydrophobic state of the surface, and the water contact angle increased from 27° to 165°. The n-octadecane coating formed on the OTS-deposited nanowires/nanowalls altered the hydrophobic state of the surface. This study provides the first demonstration that the surface wetting characteristics change from hydrophobic to hydrophilic after melting of the n-octadecane coating. In addition, this change is reversible; i.e., the hydrophilic surface becomes hydrophobic after the n-octadecane coating solidifies at the surface, and the process again occurs in the opposite direction after the n-octadecane coating melts.

The use of nanowires is of increasing interest in biomedical^1^, electronic^2^, and renewable energy^3^ applications, owing to the advantageous properties of nanowires, such as their self-assembly, high crystallinity, high surface-to-volume ratio, slow electron-hole recombination, and quantum confinement effects^4^. Silicon nanowires also have advantageous optical and structural properties that are different from those of bulk silicon^5^. Silicon nanowires created on a surface exhibit micro/nano textures, which contribute to the hydrophobicity of the surface. These hydrophobic characteristics can be utilized in self-cleaning applications. Self-cleaning surfaces are of interest in energy harvesting applications, particularly in dusty environments^6^. In recent years, climate change has triggered frequent dust storms in the Middle East, particularly in Saudi Arabia. The dust settles on surfaces and partially prevents solar radiation from reaching energy-harvesting devices, such as photovoltaic panel surfaces and volumetric receivers, which reduces device performance over time^7^. The hydrophobic characteristic of a surface is the key element for self-cleaning applications because it lowers the effort and cost of cleaning a soiled surface as compared with conventional cleaning methods^8^. Several methods have been introduced to produce silicon nanowires^9^, and some of these involve multi-step processes that require high operation precision. These processes include silicon nanowire formation via the vapor-liquid-solid (VLS) mechanism^10^, chemical vapor deposition^11^, plasma processing^12^, and chemical etching^13^. Among these methods, chemical etching is relatively simple and involves cost-effective processes^14^. The hydrophobic characteristics of a surface composed of silicon nanowires can be further enhanced via coating with low-energy silanes, such as octadecyltrichlorosilane (OTS). In addition, controlling hydrophobic characteristics locally can create a transition from the Cassie Baxter state to the Wenzel state and improve the water droplet mobility on the surface, which is essential in self-cleaning applications. Thus, it is essential to investigate the hydrophobic characteristics of OTS deposited on silicon nanowires in the presence of phase change material at the surface.

Numerous studies have been carried out to examine the physical and chemical characteristics of silicon nanowires. Minh *et al*.^15^ have investigated the passivation of oxide-free silicon nanowires via the formation of hexadecyne-derived monolayers with varying fluorine content and have demonstrated that the monolayers are stable under acidic and basic conditions. In addition, the coating provides excellent surface passivation under extreme conditions, such as ultraviolet (UV) exposure. Faro *et al*.^16^ have conducted a study on synthesizing ultrathin silicon nanowires through metal-assisted chemical wet etching and have demonstrated that the hybrid silicon nanowire/carbon nanotube system exhibits a double emission at room temperature in both the visible and infrared ranges. Dinh *et al*.^17^ have investigated the thermosensitivity of silicon nanowires induced via amorphization and have found that the thermal annealing of silicon nanowires results in amorphous structures with improved electrical conductivity. Hemed *et al*.^18^ have investigated the functionalization of silicon nanowires forming a self-assembled monolayer and have demonstrated that the morphology of the nanowires is composed of a polycrystalline core wrapped in a hydrogenated amorphous silicon (α-Si:H) shell. Hamidinezhad^19^ has examined the effect of the thickness of the catalyst layer on the morphology of silicon nanowires and has demonstrated that the diameter of the nanowires increases with increasing gold catalyst layer thickness and that the features of the nanowires are dependent on the thickness of the gold layer. Lamrani *et al*.^20^ have conducted a study on the properties of permalloy films electroplated on silicon nanowires and have shown that the formation rate of nanowires increases with dopant concentration and that the use of silicon-on-insulator samples results in a marked loss in the starting device layer under conventional process conditions, thus limiting the maximum achievable length of the nanowires. Khan *et al*.^21^ have introduced a novel approach for the fabrication of buried contact silicon nanowire solar cells with improved performance. They have used self-aligned single-step lithography to fabricate nanowire-based buried solar cells (SiNWBCs) and have demonstrated that the effectiveness of the SiNWBCs is high, with an improvement of ∼7.82% compared with that of the control selective silicon nanowire cell. Liu *et al*.^22^ have studied the effects of surface roughness on the elastic limit of silicon nanowires. They have shown that the presence of rough surfaces significantly reduces the initial elastic limit of silicon nanowires. In addition, the yield stress of rough wires with a certain cross-sectional size depends on the surface notch depth. Lajvardi *et al*.^23^ have investigated silicon nanowires synthesized via silver-assisted chemical etching. They have demonstrated that an increase in the etching time reduces the total reflectance and have observed broadband visible photoluminescence associated with the silicon nanocrystals. Jian *et al*.^24^ have studied the growth and morphology modulation of needle-like silicon nanowires and have found that the morphology of silicon nanowires can be effectively modulated by tuning the size of catalyst droplets (needle-like silicon nanowires), owing to the reduction and consumption of Si-Au catalyst droplets. Noor and Krull^25^ have presented a review on silicon nanowires for biosensing applications. They have provided physical insight into various methods for the surface functionalization of silicon nanowires and the analytical performance of silicon nanowires field emission sensors. Al-Taay *et al*.^26^ have characterized silicon nanowires catalyzed by zinc metal through a pulsed plasma-enhanced chemical vapor deposition technique. Their findings have revealed that zinc metal deposition exhibits a sharp Raman peak that corresponds to the first-order transverse optical phonon mode; in contrast, the other samples analyzed produced silicon nanowires with a broad Raman band. Coffinier and Boukherroub^27^ have modified the surface of semiconducting silicon nanowires for biosensing applications. They have demonstrated that surface functionalization allows for the controlled immobilization of biomolecules on silicon nanowires, which is important in designing selective and sensitive biosensors.

Previous studies have also investigated the hydrophobic characteristics of silicon nanowires^28^,^29^,^30^,^31^,^32^. Laminack *et al*.^28^ have considered synthesizing nanoscale silicon to accomplish the transformation from hydrophilicity to hydrophobicity. They have developed a system using variations in the surface Si:O ratios, in which the transition from hydrophilic to hydrophobic characteristics is associated with the ability to adsorb water. Ganta *et al*.^29^ have examined hydrophobic recovery in ultrathin PDMS-coated long and short silicon nanowires. They have reported that coating silicon nanowires with a few nanometers of polydimethylsiloxane (PDMS) via plasma treatment results in a ~30 ± 1° change in the superhydrophobic contact angle. Yu *et al*.^30^ have examined the superhydrophobic characteristics of carbon nanotube/silicon carbide nanowires and have shown that the composite film exhibits excellent intrinsic superhydrophobic characteristics without any functionalization. Li *et al*.^31^ have studied the fabrication of a silicon pyramid/nanowire binary structure with superhydrophobicity and have found that the binary roughness of a pyramid/nanowire structure produces a stable composite interface of silicon–air–water, and the resulting texture is responsible for the superhydrophobic characteristics of the surface. Egatz-Gomez *et al*.^32^ have investigated the hydrophobic properties of silicon nanowires and polyethylene superhydrophobic surfaces. They have demonstrated that silicon nanowires coated with low-density polyethylene result in nano and microscale surface roughness, which enhances droplet movement on the surface. The hydrophobic properties of silicon nanowires, produced via chemical vapor deposited carbon nano-flakes, have been investigated by Banerjee *et al*.^33^. They have showed that significant improvement was possible for the emission characteristics after carbon flakes with turn-on field downshifted from 9.30 to 2.77 V/μm. This behavior is related to enhancement of surface roughness, which is favorable band bending for electron emission and overall reduction of potential barrier on application of external electric field. Optical, electrical and morphological characteristics of functionalized silicon nanowires/conjugated polymer hybrid solar cells have been studied by Chehata *et al*.^34^. They have indicated that due to charge transfer efficiency, improved electrical coupling between silicon nanowires and poly[2-methoxy-5-(2′-ethylhexyloxy)-1,4-phenylene vinylene] takes place. The performance of photovoltaic device shows a significant improvement with the progressive addition of polystyrene-silicon nanowires. Li *et al*.^35^ has investigated capacitive sensing characteristics of silicon nanowires. They demonstrated that the humidity sensors have the simple structure and the high performance such as the high sensitivity, the wide humidity detection range, the good stability, and repeatability. A study of fabrication and characterization of polycrystalline silicon nanowires has been carried out by Sun *et al*.^36^. They have shown that a superhydrophobic surface is possible on the as-etched silicon nanowire arrays without surface modification with any organic low-surface-energy materials. Wang *et al*.^37^ examined the fabrication of silicon nanowire arrays with controllable dimensions. They have demonstrated that adjusting the wet-etching time, the length of silicon nanowires can be controllable. In this case, the silicon nanowire arrays exhibit ultralow reflectance and interesting wettability that are of great importance to photovoltaics and thermal management applications.

Although the surface characteristics, including hydrophobicity, of silicon nanowires have been previously studied^28^,^29^,^30^,^31^,^32^, those studies have not focused on the modification of hydrophobic surface characteristics. The present study considers the surface characteristics, including hydrophobicity, of silicon nanowires. A two-step chemical etching process was used. In the first step, HF/AgNO_3_ 5 M/0.02 N was used, and in the second step, HF/H_2_O_2_ 5 M/30% etchant was incorporated. OTS was deposited onto the silicon nanowire surfaces to improve the surface hydrophobicity. The treated surfaces of the silicon nanowires were then coated with a thin layer of n-octadecane phase change material. Because liquid n-octadecane has a high surface energy^38^, it covers the treated silicon nanowire surface once it has melted. This in turn decreases the hydrophobic characteristics of the surface. Consequently, locally scattered hydrophobic regions are generated on the surface. The reversible hydrophobic state of the regions coated with n-octadecane was investigated. The change in hydrophobic state when n-octadecane was melted and solidified was examined, as were the friction coefficient and UV-visible reflectivity of the treated silicon nanowires surfaces.

## Experimental

P-type <100> silicon wafers with an electrical resistivity of 1–10 ohm cm were used to form silicon nanowires. A metal-assisted chemical etching method was used, and AgNO_3_ (99.8%), H_2_O_2_ (30% in water), H_2_SO_4_ 98%, and HF (48%) were used as the chemical etchants. The silicon wafers were cleaned with acetone, isopropanol, and H_2_O for 15 min each, and this was followed by a piranha solution of H_2_O_2_:H_2_SO_4_ (1:1 v/v) for 10 min. After cleaning, the wafers were thoroughly rinsed and placed in an electrodeionization system (EDI) before being dried with nitrogen. The samples were then directly introduced into the HF/AgNO_3_ solution at different concentrations for 1 min. After this process, the silicon wafers were covered with Ag nanoparticles. The wafers were then immersed in a second etching solution of HF/H_2_O_2_ 5 M/30% etchant, and this was followed by EDI rinsing and drying with nitrogen. To remove the residual Ag from the silicon nanowires, the samples were immersed in a mixture H_2_O:HCl:HNO_3_ (1:1:1 v/v/v) for one hour, and this was again followed by EDI rinsing and drying with nitrogen.

The surface morphologies and textures of the as-synthesized samples were characterized with a focused ion beam (FIB) field emission dual beam scanning electron microscope (FESSEM) and atomic force microscope (AFM). The AFM tip was made of silicon nitride probes (*r* = 20–60 nm) with a manufacturer-specified force constant, *k*, of 0.12 N/m. The crystalline structures of the silicon nanowires were characterized by X-ray diffraction (XRD). TEM images were obtained using TEM Philips CM20 operating at 200 kV equipped with “Megaview” charged coupled device camera from SIS. X-ray photoelectron spectrometer (XPS) was performed incorporating ESCALAB 220 XL spectrometer. A monochromatic Al K_α_ X-ray source (1486.6 eV) was operated in the constant analyzer energy mode (CAE = 100 eV for survey spectra and CAE = 40 eV for high resolution spectra). The surface reflectivity was measured with a UV-visible spectrophotometer (UV-2600 Shimadzu).

The friction coefficient of the as-synthesized sample surfaces was measured using a scratch testing facility. A linear micro-scratch tester (MCTX-S/N: 01-04300) was used. The scratch tester was set at a contact load of 0.03 N and an end load of 5 N. The scanning speed was set to 1 mm/min with a loading rate of 5 N/s, thus resulting in a total scratch length of 1 mm.

A goniometer (Kyowa, model DM 501) was used to conduct sessile drop tests for the measurement of the droplet contact angle. Deionized water was used in the sessile drop experiments, and the droplet volume was controlled with an automatic dispensing system. The images of the droplets were taken one second after deposition of the water droplet on the surface.

## Results and Discussion

Chemical etching of the p-type Si <100> wafer was used to produce silicon nanowires at the wafer surface. To improve the silicon nanowire surfaces’ surface hydrophobicity, the surfaces were treated through deposition of OTS. To generate local hydrophilic sites at the surface, the treated silicon nanowire surfaces were coated with n-octadecane phase change material. The surface hydrophobicity switched to hydrophilicity after the phase change material underwent melting at the surface. The surface morphology and texture were examined using SEM and AFM. The scratch tests and UV-visible reflectivity tests were performed before and after the silicon nanowire surfaces were treated. The surface hydrophobicity was assessed through the water contact angle measurement method.

**Figure 1:**
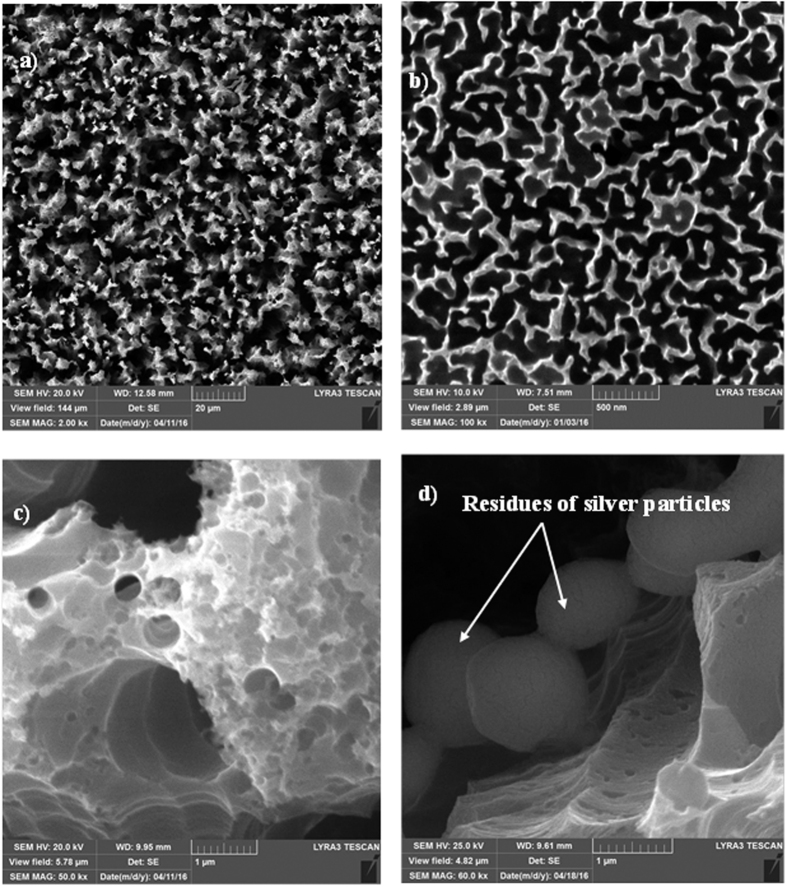
SEM micrographs of the silicon nanowire/nanowalls: (**a**) silicon nanowires/nanowalls, (**b**) close-up view of the silicon nanowire/nanowalls and the connection of the silicon nanowires forming nanowalls, (**c**) porous structures in the region of the nanowall’s tip, (**d**) residues of silver particles at the nanowall wall.

Figure (1) shows SEM micrographs of the silicon nanowires produced from the silicon wafer. The silicon nanowires were nearly uniformly distributed at the surface of the silicon wafer. The tips of the silicon nanowires were partially connected, forming open cell-like structures (Fig. 1(a,b)). A similar texture has also been reported in a previous study^23^. The formation of open cell-like structures is associated with the etching time and etchant concentration^39^. In this case, a thin layer of silver was preferentially oxidized and anisotropically etched in an aqueous solution containing HF and an oxidant (H_2_O_2_, O_2_ present in H_2_O) while some of the bare silicon regions were left unetched. This feature appeared as silicon nanowalls connecting to silicon nanowires. Several etching tests were carried out for various etchant concentrations and durations to optimize the depth and spacing of the silicon nanowires. In some regions, the tips are loose and not connected to other nanowires. The spacing between the silicon nanowire tips was critical to achieve a surface topology with hydrophobic surface characteristics. The spacing, as measured from the SEM micrograph (Fig. (1b)), was approximately 125 nm, which represents closely spaced nanowires and nanowalls. Consequently, the surface texture exhibited hydrophilic characteristics. Close examination of the SEM micrographs revealed that some porous structures formed at the surface (Fig. (1c)). The pore-like structures were identified as tiny holes a few nanometers in diameter. The formation of nanopores is associated with one or all of the following: i) silicon etching is catalyzed by silver particles; in this case, the large silver particles produced holes, and small silver particles produced nanopores; and ii) holes are generated during the HF/NO_3_^−^ chemical etching process^40^,^41^. The presence of residual silver particles was evident, as shown in Fig. (1d) even though these particles were removed from the surface after the etching. The silicon nanowires were produced through initial etching with 5 M HF 0.02 N AgNO_3_ for 1 minute and subsequent etching for 10 minutes with HF/H_2_O_2_ 5 M/30% in H_2_O. Consequently, the formation of the porous structures was initiated during the first etching and the structures were further developed during the second etching process. Nevertheless, the coverage area of the porous structures was limited to the tip region of the connected nanowires (nanowalls).

**Figure 2:**
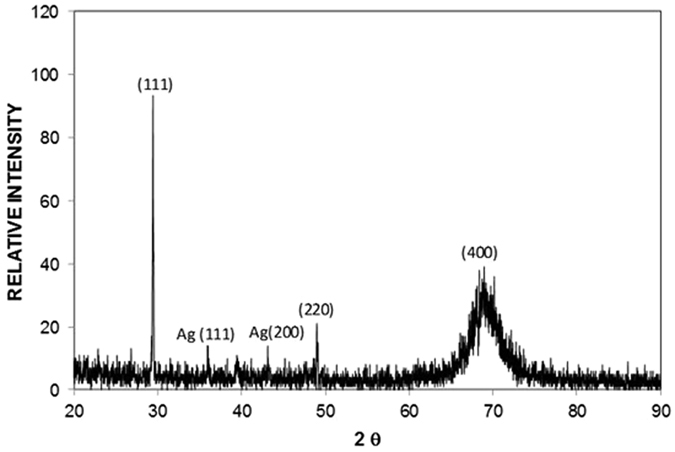
X-ray diffractogram of the nanowires/nanowalls surface.

**Figure 3:**
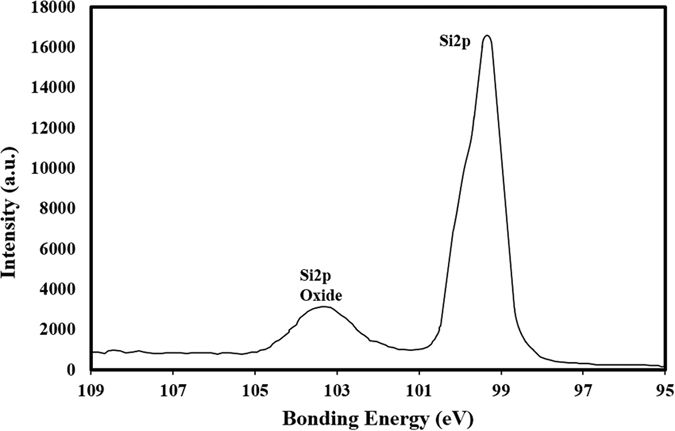
XPS data for silicon nanowire. Overlapping of Sip_2/3_&Sip_1/3_ represents silicon peak while Si2p corresponds to silicon oxide peak.

**Figure 4:**
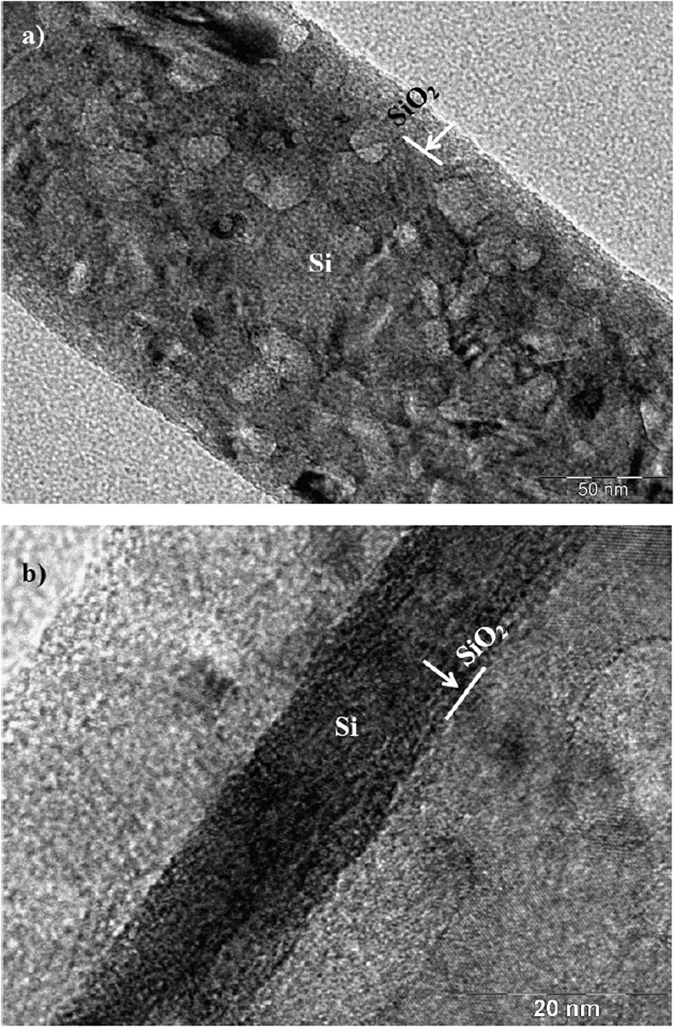
TEM images of silicon nanowire: (**a**) large diameter size silicon nanowire with presence of SiO_2_ cover around the silicon core, and (**b**) small diameter silicon nanowire and small size SiO_2_ cover around the silicon core.

**Figure 5:**
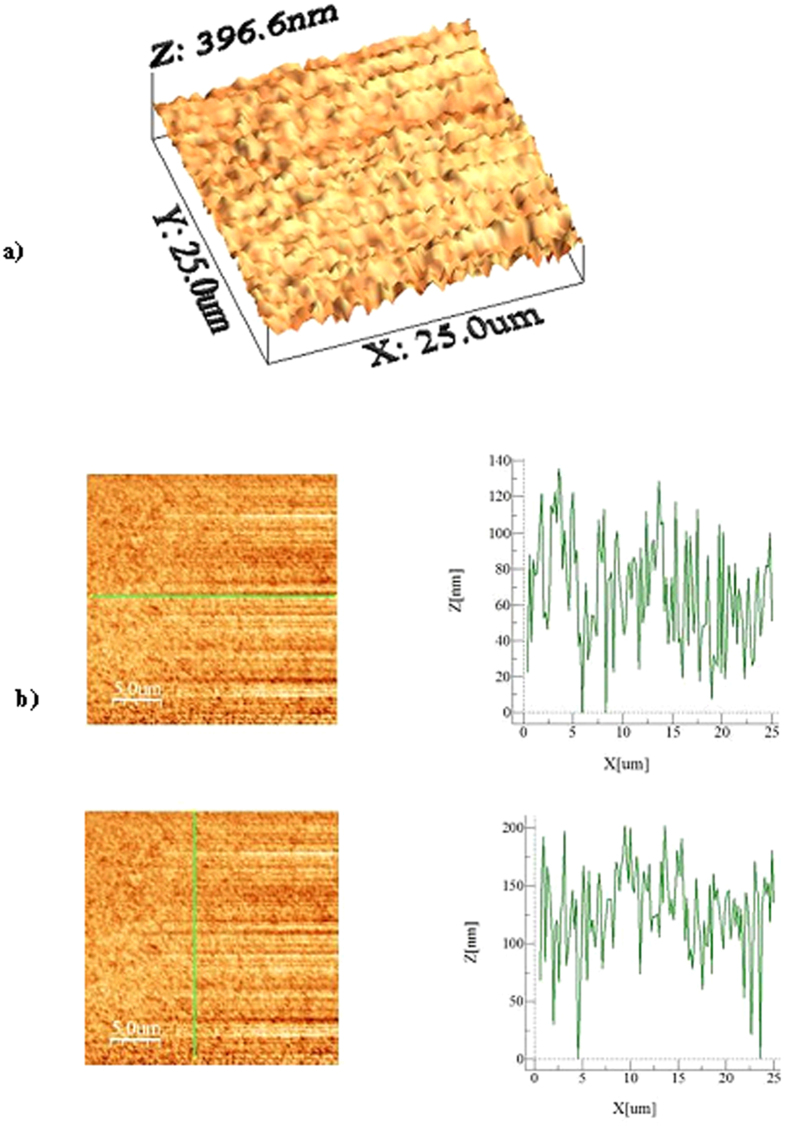
Atomic force microscope images of silicon nanowires surface: (**a**) 3-dimensional image of the surface, and (**b**) line scan across the surface.

**Figure 6:**
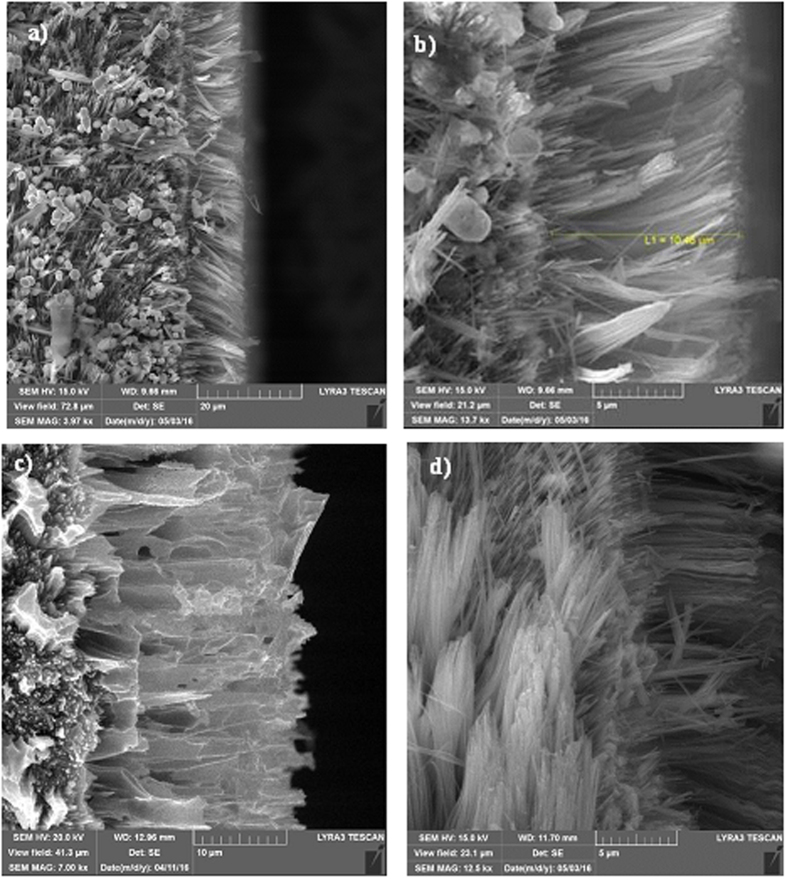
SEM micrographs of the cross section of silicon nanowires/nanowalls: (**a**) cross-section of the silicon nanowires and the presence of silver particles at the bottom surface, (**b**) silicon nanowires/nanowalls with a changing width toward the tip, (**c**) silicon nanowires with connected tips, (**d**) loose ends of the silicon nanowires.

Figure (2) shows an X-ray diffractogram of the silicon nanowires/nanowalls on the silicon wafer surface. The silicon diffraction peaks appeared at 28.4° (111), 47.3° (220), and 69.27° (400). These peaks showed the crystalline silicon phases, which are commonly observed for silicon nanowires^26^. In addition, the silver peaks appeared at 38.32° (111) and 44.50° (200). The presence of silver peaks was associated with the residual of silver particles after the etching (Fig. (1d)). Figure (3) shows XPS data for the silicon nanowires/nanowalls on the silicon wafer surface. XPS data demonstrates two peaks of Si 2p spectra. The first peak, which corresponds to the lower binding energy, at 99.71 eV corresponds to silicon and the other peak, which is associated with the higher binding energy, at 103.77 eV is due to silicon oxide. The presence of silicon oxide peak is related to the oxidation taking place during the etching process. It should be noted that the etching occurs along the vertical direction relative to the surface of the substrate in the solutions. In this case, the completion of the hole injection oxidation and mass transport (dissolution) with respect the surface atom density becomes important, which is consistent with the previous findings^42^. The silicon 2p oxide line intensity increases with the increase in the oxide thickness; in which case, the silicon 2p line intensity decreases^43^. Figure (4) shows TEM images of silicon nanowires. The TEM image of the silicon nanowire demonstrates a crystalline silicon core with surrounding of a thin layer of an amorphous silicon oxide (Fig. (4a)). The silicon core diameter is in the order of 130 nm while the average silicon oxide shell thickness is in the order of 8 nm. However, some small variations in silicon oxide thickness are observed, which is probably linked to the orientation of the silicon crystals in the silicon nanowire core. During the process of metal-assisted chemical etching at high temperatures, silver nanoparticles disintegrate (diffuse out) and sink into nanowire surfaces, which possible cause secondary etching on the sidewalls while forming pit sites on nanowires^44^. However, this situation is not observed on the TEM images. The silicon/silicon oxide interface as shown in Fig. (4a) is sharp demonstrating no phase mixing. In the case of Fig. (4b), silicon nanowire diameter reduces, which is in the order of 12 nm with a very fine silicon oxide layer around the outer surface. Therefore, the growth process works equally well to form the nanowire sizes ranging from 12 nm to 130 nm. Figure (5) shows the 3-dimensional AFM image of the silicon nanowire surface (Fig. (5a)) and a line scan (Fig. (5b)) of the surface decorated by nanowires. The surface texture was composed of nearly equally distributed pillars that were closely spaced. This result was also visible from the line scans (Fig. (5b)). In this case, spikes in the curve represented the presence of nanowires at the surface. The average surface roughness was estimated to be approximately 210 nm. The average roughness was based on the surface texture, as captured by the AFM tip operating in tapping mode. Figure (6) shows SEM micrographs of cross-sections of silicon nanowires produced at the silicon wafer surface. The depth of the nanowires extended uniformly below the surface. The average height of the silicon nanowires was approximately 10.5 μm (Fig. (6a)). In general, the nanowires extended from the surface in a parallel manner. However, the width of the nanowires decreased toward the tips (Fig. (6b)), and this decrease was associated with the penetration of the etchant and the high rate of etching in the region of the tip section. In addition, we observed nanowalls among the nanowires (Fig. (6c)), which resulted from the joining of nanowires. However, non-connected nanowires were also observed in the SEM micrographs (Fig. (6d)).

**Figure 7:**
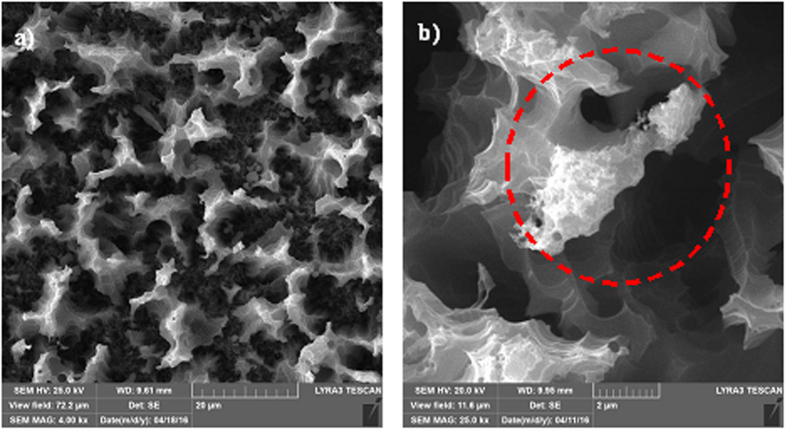
SEM micrographs of OTS deposited on the silicon nanowire/nanowall surfaces: (**a**) OTS-coated top surface and (**b**) close-up view of the OTS deposition at the surface circled in red.

**Figure 8:**
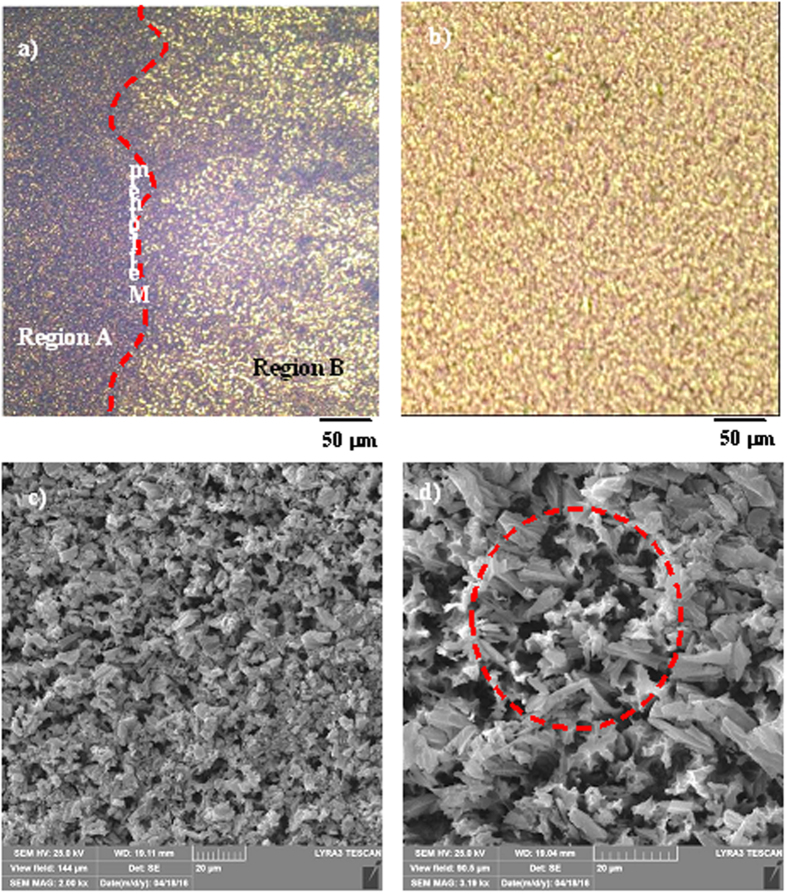
Optical images and SEM micrographs of OTS-coated silicon nanowires/nanowalls with the n-octadecane coating at the surface: (**a**) Optical image of the liquid-solid interface of the n-octadecane and the melt isotherm marked with a red line, (**b**) optical image of the liquid phase of n-octadecane, (**c**) SEM micrograph of the solid phase of n-octadecane, and (**d**) solid phase of n-octadecane with locally exposed OTS. Some OTS regions exposed to air are shown in the red circle.

Figure (7) shows SEM micrographs of the surface of the nanowires after OTS deposition and n-octadecane coating. In the case of OTS- and n-octadecane-deposited surfaces, OTS was deposited first, and this was followed by coating with n-octadecane. A thin film of OTS was deposited on the nanowire surfaces; therefore, its presence appeared to be at the tip regions of nanowires and nanowalls (Fig. (7a)). Because the immersion and pulling speeds of the silicon wafer with nanowires at the surface were approximately 5 mm/s, the OTS deposition at the surface formed a thin layer, estimated to be a few nanometers thick. Close examination of the SEM micrographs (Fig. (7b)) revealed the presence of a thin layer of OTS in the tip section of the nanowires/nanowalls. OTS deposits were not observed between nanowires/nanowalls; consequently, only the tip section of the nanowires/nanowalls was covered by the OTS depositions. Moreover, Fig. (8) shows optical and SEM micrographs of the OTS-deposited and n-octadecane-coated surface. The n-octadecane coating was deposited via a dip coating technique by using the liquid phase of n-octadecane. Because the solidus temperature of n-octadecane is 301 K, it remains in the solid phase at temperatures lower than 301 K^45^. Hence, n-octadecane remained in the solid phase at room temperature once the coating was complete. Although coating in the liquid phase formed a continuous film at the nanowire surfaces (Fig. 8(a,b)), the solidified n-octadecane exhibited solid granule-like structures at the nanowire surfaces, as shown in Fig. 8(c,d). The size of the granule-like structure was approximately 5 μm. In addition, liquid n-octadecane completely wetted the surface; however, after n-octadecane solidified at room temperature (293 K), the surface area covered by n-octadecane became small because the density difference between the solid and liquid phases leads to a decrease in volume upon solidification^45^. This decrease in volume gave rise to partial coverage of the nanowire surfaces by n-octadecane granules. Consequently, some of the OTS-coated nanowire/nanowall surfaces became visible, as observed in the SEM micrograph (Fig. (8d)). However, the uncovered region was locally scattered and did not form a clear pattern on the surface.

**Figure 9:**
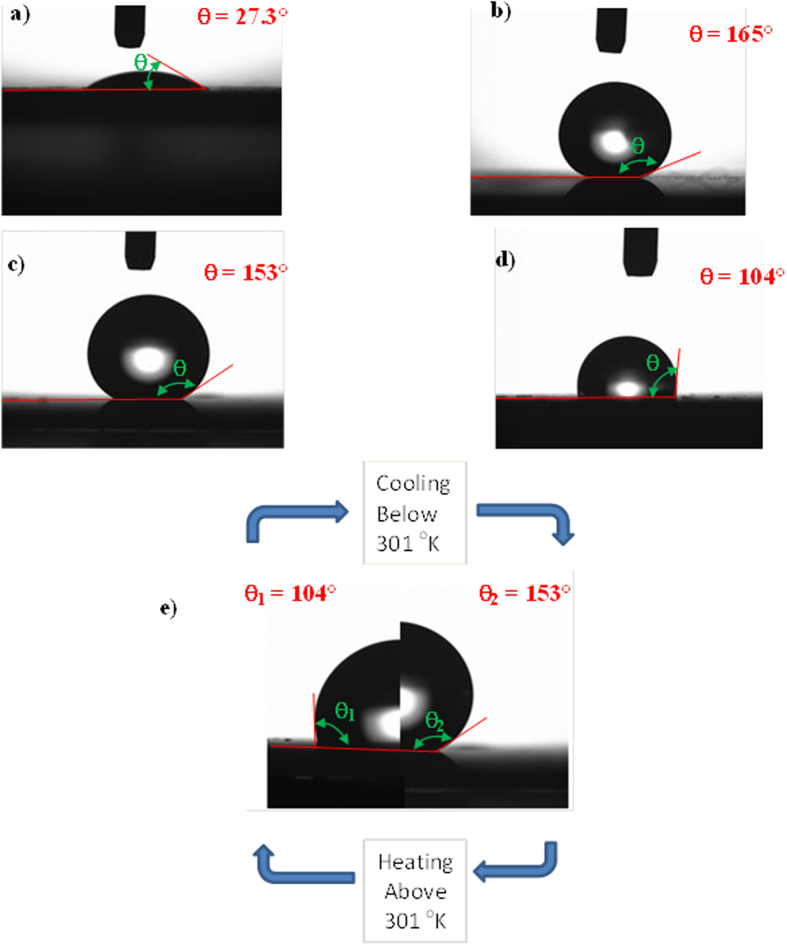
Contact angles of droplets on: (**a**) silicon nanowires/nanowalls, (**b**) OTS-coated silicon nanowires/nanowalls, (**c**) OTS-coated silicon nanowires/nanowalls with solid-phase n-octadecane at the surface, and (**d**) OTS-coated silicon nanowires/nanowalls with liquid-phase n-octadecane at the surface, (**e**) reversible contact angles from hydrophobic to hydrophilic due to the phase change of n-octadecane.

Figure (9) shows the water droplet images on the silicon wafers featuring nanowires/nanowalls at the surface, the OTS-coated nanowires/nanowall surfaces, and the OTS-deposited and n-octadecane-coated nanowire/nanowall surfaces. Table 1 provides the corresponding contact angles and contact angle hysteresis. The wetting characteristics of the surfaces differed significantly for silicon nanowires/nanowalls (Fig. (9a)) compared with bulk silicon. The nanowire/nanowall surfaces exhibited hydrophilic characteristics with a low contact angle compared with the unetched silicon surface. These hydrophilic characteristics were attributed to the closely spaced nanowires/nanowalls at the surface, which did not yield a sufficient number of air gaps to satisfy the Cassie and Baxter state. However, OTS deposition significantly lowers the surface energy (15.36 mN/m^46^), and this in turn enhanced the surface hydrophobicity. In this case, the contact angle increased to 165.7° after OTS deposition (Fig. (9b)). OTS deposition occurred on the nanowire/nanowall surfaces (Fig. (7a)). In the case of the n-octadecane-coated and OTS-deposited nanowire/nanowall surfaces, the surface exhibited either hydrophobic or hydrophilic characteristics depending on whether n-octadecane was in the solid or liquid phase (Fig. 9(c,d)). The liquid phase of n-octadecane resulted in a water contact angle of approximately 104 ± 5°, which was on the border between hydrophobic and hydrophilic. The liquid phase of the n-octadecane surface is called hydrophilic for simplicity. A possible explanation for the hydrophilic characteristics of the n-octadecane-coated surface is related to the large coverage area of the liquid phase of n-octadecane on the surface. The spreading rate of the liquid phase is high because of the high surface energy of 27.4 mN/m^47^. Thus, it entirely covered the OTS coating at the nanowire/nanowall surfaces while giving rise to hydrophilic surface characteristics (Fig. 8(a,b)). The liquid film thickness of n-octadecane was measured using an ellipsometer to be approximately 1.5 μm. This result indicated that a stable liquid film of n-octadecane was formed while the entire surface was wetted (Fig. 8(a,b)). However, the surface became hydrophobic after solidification of the n-octadecane film (Fig. (9c)), because of the granule-like structures formed by the solid n-octadecane at the OTS-deposited nanowire/nanowall surfaces. This, in turn, enabled a portion of the OTS-deposited surface to become exposed to air (Fig. (6d)). The n-octadecane coating was melted and solidified, and the same surface hydrophobic characteristics were observed. Thus, surface hydrophobicity can be changed reversibly on the n-octadecane- and OTS-coated nanowire/nanowall surfaces. The constant temperature heating at 308 K was applied at the end of the silicon wafer with nanowires/nanowalls at the surface. The contact angle of the water droplet located at the melt-solid interface was also measured (Fig. (9d)). The contact angle was reduced by nearly 50° on the melted surface, whereas it remained high on the solid surface. Because the heating took place slowly, the phase change also took place slowly. In this case, the geometric features of the droplet differed between the liquid and solid phases, as shown in Fig. (9e).

**Figure 10:**
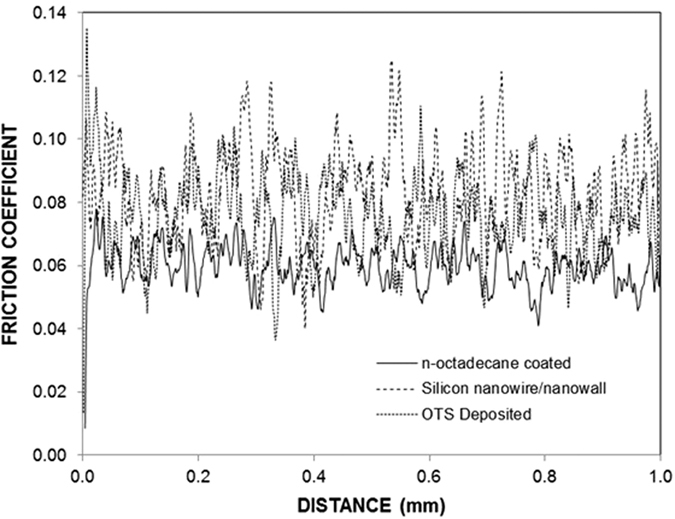
Friction coefficients of the silicon nanowires/nanowalls, the OTS-deposited silicon nanowires/nanowalls, and the OTS-deposited silicon nanowires/nanowalls with the n-octadecane coating.

**Figure 11:**
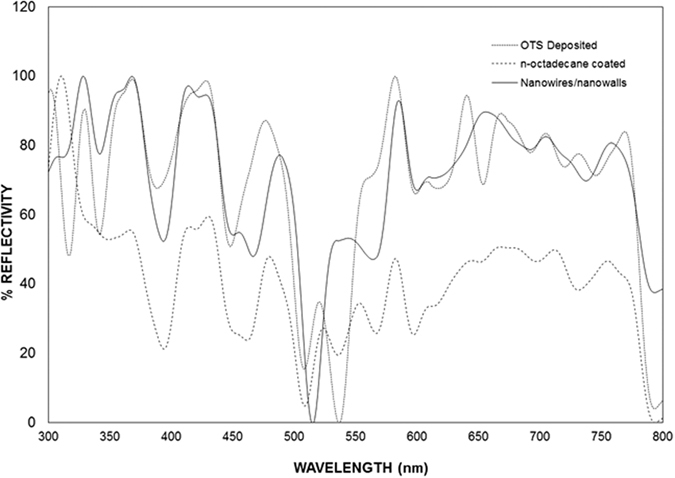
Reflectivities of the silicon nanowire/nanowalls, the OTS-deposited silicon nanowire/nanowalls, and the OTS-deposited silicon nanowire/nanowalls with n-octadecane in the solid phase.

Figure (10) shows the friction coefficient obtained from the scratch tests for a silicon wafer with nanowires/nanowalls at the surface and for OTS-deposited and n-octadecane-coated nanowires/nanowalls. The average friction coefficient was approximately 0.09 for the nanowire/nanowall surfaces, and it oscillated along the scratch length. This result was attributed to the open cell-like structures formed at the surface, which gave rise to air gaps at the surface. In the case of the OTS-deposited and n-octadecane-coated nanowire/nanowall surfaces the average friction coefficient decreased slightly (0.07), a result associated with the lubrication characteristics of the n-octadecane. In this case, n-octadecane acted as a solid lubricant at the surface while lowering the friction coefficient. Figure (11) shows the UV-visible reflection data for the nanowire/nanowall surfaces with or without the OTS deposition and n-octadecane coating. The reflectivity was considerably different for the OTS-deposited and n-octadecane-coated nanowire/nanowall surfaces. In this case, the surface reflectivity decreased by over 30% for the wavelengths ≤500 nm and wavelengths ≥550 nm. In the case of OTS-deposited nanowire/nanowall surfaces, the reflectivity changed slightly compared with the uncoated nanowire/nanowall surfaces. Consequently, surface reflectivity is influenced by the n-octadecane coating at the surface.

## Conclusion

Silicon nanowires/nanowalls on a p-type Si <100> silicon wafer were produced using a metal assisted chemical etching method. OTS was deposited onto the nanowire/nanowall surface using a dip coating method to enhance the surface hydrophobicity. The immersion and pulling speeds were set to 5 mm/s to form a thin OTS deposition layer on the surface. This is the first time that a phase change material on the surfaces of OTS deposited nanowires/nanowalls has been introduced to achieve a transition from a hydrophobic to hydrophilic state on the surface. A dip coating method was adopted to form a 1.5-μm-thick liquid n-octadecane layer on the surfaces of the silicon nanowires/nanowalls. The morphological and textural structures of the nanowire/nanowall surfaces were examined using SEM and AFM. The surface hydrophobic states of the resulting surfaces were assessed through the sessile water droplet contact angle measurement method. Scratch tests were performed to measure the friction coefficient of the surfaces, and UV-visible reflectivity tests were performed to determine the effects of OTS deposition and n-octadecane coating on the reflectivity of the silicon wafers with nanowires/nanowalls. Chemical etching resulted in the formation of vertically oriented silicon nanowires at the silicon surface. The length of the nanowires extended approximately 10.5 μm from the surface. Nanowires formed open cell-like structures, and cell walls appeared in the form of nanowalls. Consequently, the chemically etched surfaces comprised nanowires and nanowalls. The average surface roughness of the silicon nanowires/nanowalls, as determined from the AFM data, was approximately 210 nm. The surfaces of the nanowires/nanowalls exhibited hydrophilic characteristics; however, the deposition of OTS at the surface changed the surface state to hydrophobic. The water droplet contact angle of the nanowire/nanowall surfaces was approximately 28°, and it increased to 165° after OTS deposition. Although OTS deposition occurred in the regions close to the tips of the nanowires/nanowalls, it influenced the surface hydrophobic characteristics significantly. The n-octadecane coating on the OTS-deposited surface also influenced the hydrophobic state of the nanowire/nanowall surface. In this case, the high surface energy of the liquid phase n-octadecane lowered the water contact angle of the surface to approximately 104°. The water droplet contact angle increased to approximately 150° after solidification of the n-octadecane coating layer. This behavior was attributed to the formation of granule-like structures after the solidification of n-octadecane at the surface. The formation of these structures caused a portion of the OTS-deposited surface to become exposed to air. Moreover, several tests were performed to assess the reversibility of the surface hydrophobic states before and after the phase change of n-octadecane. This is the first study to demonstrate that the hydrophobic state of the surface is reversible after n-octadecane solidifies. The n-octadecane coating alters the friction coefficient at the surface; the presence of the n-octadecane coating acts as a solid lubricant while slightly lowering the surface friction coefficient. In addition, the n-octadecane coating lowers the reflectivity of OTS coated nanowire/nanowall surfaces by nearly 30%.

## Additional Information

**How to cite this article**: Yilbas, B. S. *et al*. Surface Characteristics of Silicon Nanowires/Nanowalls Subjected to Octadecyltrichlorosilane Deposition and n-octadecane Coating. *Sci. Rep.* **6**, 38678; doi: 10.1038/srep38678 (2016).

**Publisher's note:** Springer Nature remains neutral with regard to jurisdictional claims in published maps and institutional affiliations.

## Figures and Tables

**Table 1:** Water droplet contact angle and hysteresis for the silicon nanowires/nanowalls, the OTS-deposited silicon nanowires/nanowalls, and the silicon nanowires/nanowalls with n-octadecane present on the surface.

	Nanowires/Nanowalls	OTS Deposited	n-octadecane Coated – Solid Phase	n-octadecane Coated – Liquid Phase
Contact Angle (Degrees)	27.3 ± 2	165 ± 5	155 ± 5	104 ± 5
Contact Angle Hysteresis (Degrees)	N/A	1	12	N/A

N/A indicates not measured with accuracy.
